# The Diagnostic Value of qPCR Quantification of *Paenibacillus larvae* in Hive Debris and Adult Bees for Predicting the Onset of American Foulbrood

**DOI:** 10.3390/vetsci11090442

**Published:** 2024-09-20

**Authors:** Bojan Papić, Lucija Žvokelj, Metka Pislak Ocepek, Barbara Hočevar, Monika Kozar, Rene Rus, Urška Zajc, Darja Kušar

**Affiliations:** 1 Institute of Microbiology and Parasitology, Veterinary Faculty, University of Ljubljana, Gerbičeva 60, 1000 Ljubljana, Slovenia; 2 Institute of Pathology, Wild Animals, Fish and Bees, Veterinary Faculty, University of Ljubljana, Gerbičeva 60, 1000 Ljubljana, Slovenia

**Keywords:** *Paenibacillus larvae*, American foulbrood, quantitative PCR, logistic regression

## Abstract

**Simple Summary:**

An increased *Paenibacillus larvae* count in hive material is correlated with an increased risk of developing American foulbrood (AFB), a serious disease of the honeybee brood. In this study, we quantified *P. larvae* in adult bee and hive debris samples using quantitative PCR. *P. larvae* count was analyzed with respect to the presence and intensity of AFB clinical signs to evaluate the diagnostic value of both sample types. A threshold was established for the identification of apiaries with increased risk of developing AFB, expressed as *P. larvae* cell count per milliliter of hive debris. *P. larvae* counts were significantly lower in AFB-unaffected colonies than in AFB-affected colonies. Both sample types showed a comparatively high diagnostic value for the prediction of AFB based on the *P. larvae* count. These results help to improve the early detection of high-risk colonies by understanding the correlation between *P. larvae* counts and the occurrence of AFB. When followed by appropriate sanitary measures, early detection of AFB is crucial to prevent the outbreak and further spread of AFB as well as to minimize colony losses and associated costs to beekeepers and pollination.

**Abstract:**

American foulbrood (AFB) is a serious infectious disease of honeybees (*Apis mellifera*) caused by *Paenibacillus larvae*. Increased *P. larvae* count in hive-related material is associated with an increased risk of AFB. Here, we quantified *P. larvae* cells in 106 adult bee and 97 hive debris samples using quantitative PCR (qPCR); 66/106 adult bee and 66/97 hive debris samples were collected simultaneously from the same bee colony (paired-sample design). The corresponding bee colonies were also examined for the presence of AFB clinical signs. A binary logistic regression model to distinguish between AFB-affected and unaffected honeybee colonies showed a strong diagnostic accuracy of both sample types for predicting the onset of AFB based on *P. larvae* counts determined by qPCR. The colonies with a *P. larvae* count greater than 4.5 log cells/adult bee or 7.3 log cells/mL hive debris had a 50% probability of being clinically affected and were categorized as high-risk. The AFB-unaffected colonies had significantly lower *P. larvae* counts than the AFB-affected colonies, but the latter did not differ significantly in *P. larvae* counts in relation to the severity of clinical signs. Both bee-related sample types had a high diagnostic value for predicting disease outcome based on *P. larvae* counts. These results improve the understanding of the relationship between *P. larvae* counts and AFB occurrence, which is essential for early detection of high-risk colonies.

## 1. Introduction

American foulbrood (AFB), caused by the spore-forming bacterium *Paenibacillus larvae*, is one of the most devastating diseases of honeybees [1]. Honeybee larvae become infected by ingesting only a few spores of *P. larvae* present in contaminated food [2]. In the European Union, AFB is on the list of diseases (Commission Delegated Regulation 2018/1629) for which specific restrictions are provided, including immediate notification to the competent veterinary authority (EU Animal Health Law 2016/429) [3,4]. In Slovenia, the national regulation requires that all apiaries within the affected 3-km radius (i.e., AFB zone) around the AFB-affected apiary should be examined for the presence of AFB [5]. If a new affected apiary is identified, the AFB zone is extended accordingly. AFB is diagnosed when characteristic clinical signs are identified in the honeybee colony and *P. larvae* is confirmed in the bee-related samples by culture and/or molecular methods. All positive colonies must be destroyed or the technique of artificial swarming (shaking) is used. In addition, restrictions in bee trade are imposed in the AFB zone, resulting in significant economic losses to the beekeeping industry.

Colonies with an elevated spore count are more likely to develop AFB clinical signs [6,7,8,9]. Therefore, reliable and cost-effective methods to quantify *P. larvae* in bee-related samples are needed. Two commonly used approaches for quantifying *P. larvae* are culture-based plate counting and quantitative PCR (qPCR). Several culture-based studies have shown a strong positive association between AFB occurrence and increased *P. larvae* counts in bee-related samples, namely honey, adult bees, hive debris and powdered sugar collected after 20–25 min from a sheet of paper placed at the bottom of the hive [6,7,8,9,10,11,12,13,14,15,16]. Cultivation of *P. larvae* spores from bee-related samples suffers from two important limitations: poor and inconsistent spore germination and overgrowth by other bacterial contaminants (concomitant sporobiota) [8,16]. In addition, it is more time-consuming and requires species identification [17]. The qPCR-based quantification of *P. larvae* is superior in sensitivity and turnaround time to cultivation, is more easily applicable to various bee-related sample types and does not require an additional species identification step [8]. It is therefore a promising method for AFB surveillance to identify colonies with high risk of soon developing clinical signs of AFB. Such apiaries should undergo thorough clinical examinations by bee health specialists and targeted control measures should be implemented. Shaking bees into a new hive with new frames and foundations (known as the shook swarm method) is believed to be a very efficient AFB control measure in clinically unaffected colonies with increased likelihood of developing the disease [18,19]. General good beekeeping practices such as thorough disinfection/incineration of the beekeeping equipment should also be followed in high-risk apiaries [18].

Only a limited number of similar studies that use qPCR-based quantification of *P. larvae* from colonies with a known AFB clinical status have been performed, namely for honey, adult bees and hive debris [8,14]. Hive debris and adult bees are both easily accessible and can be collected through non-invasive sampling. A previous study [14] reported a limited diagnostic value of qPCR for the quantification of *P. larvae* in adult bees and winter hive debris compared with the culture method, but it should be noted that such results could be due to suboptimal DNA extraction and PCR inhibitors. On the contrary, our previous study [8] showed that increased *P. larvae* count, determined by TaqMan probe-based qPCR that was developed and calibrated using digital PCR (dPCR) to enable absolute quantification of *P. larvae*, in seasonal hive debris and honey from brood combs is significantly associated with increased AFB occurrence. The constructed TaqMan assay targeted a single-copy metalloproteinase gene, showed no evidence of PCR inhibition for any of the sample types examined and was implemented according to Minimum Information for Publication of Quantitative Real-Time PCR Experiments (MIQE) guidelines [20], allowing for transparent, reproducible and accurate quantification. In this study, we compare the diagnostic value of *P. larvae* counts in seasonal hive debris and adult bees from brood combs (determined by qPCR) to predict the occurrence of AFB disease, thereby extending the application of the qPCR assay to adult bees and assessing its diagnostic value.

## 2. Materials and Methods

### 2.1. Sampling

Seasonal hive debris and adult bees from brood combs were sampled from May 2022 to July 2023. A total of 106 adult bee samples and 97 hive debris samples were collected from 20 AFB-affected apiaries within an AFB zone and 12 AFB-unaffected apiaries outside an AFB zone (Table 1 and Table S1); each sample was collected from an individual bee colony. Out of the 136 adult bee and hive debris samples from AFB-unaffected colonies, 123 samples originated from 14 different AFB-affected apiaries within an AFB zone and 13 samples originated from 12 different AFB-unaffected apiaries outside an AFB zone (Table S1). Samples from AFB-affected apiaries were collected in the scope of veterinary clinical examinations of bee colonies upon suspicion of AFB, followed by *P. larvae* cultivation from the infected brood and species identification by matrix-assisted laser desorption/ionization time-of-flight mass spectrometry (MALDI-TOF MS; Microflex LT System, Bruker Daltonics, Bremen, Germany). Disease severity in AFB-affected colonies was ranked from 1 (the mildest signs) to 3 (the most severe signs) according to the previously described scheme [8]. Disease severity in colonies without clinical signs was ranked as 0.

Sample distribution according to the clinical signs in the corresponding bee colony is shown in Table 1. A total of 66/106 adult bee and 66/97 hive debris samples were collected simultaneously from the same bee colony (paired sample design). This minimized the variability between the samples due to individual hive/colony characteristics and allowed the evaluation of the correlation between qPCR-based *P. larvae* counts in adult bees and hive debris, i.e., direct comparison of the diagnostic value of both sample types based on the receiver operating characteristic (ROC) analysis. For additional information on the samples, see Table S1.

**Figure 1:**
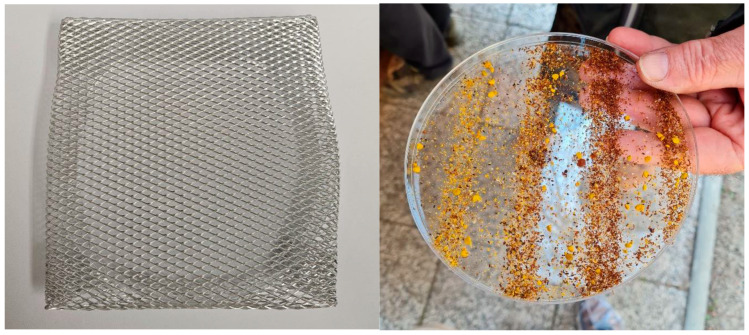
Tray for the collection of hive debris. An unused tray covered with a metal net (**left**) and a used tray containing hive debris with the metal net removed (**right**) are shown.

Both sample types were collected in sterile containers from individual honeybee colonies with known AFB status. If present in sufficient amount (5 mL), seasonal hive debris was collected during the first clinical examination (when the disease was suspected). Alternatively, sampling boards were placed at the bottom of all hives within the AFB-affected apiary during the first clinical examination to detect all potential AFB cases occurring until the first control examination (i.e., one month after the initial examination). These sampling boards consisted of a plastic collection tray covered with a wired net to prevent larger objects such as live or dead adult bees from entering the collection tray (Figure 1). During the control examination, both hive debris and adult bees were collected. A subset of samples was then examined by including at least one colony for each degree of disease severity. Hive debris and adult bees from apiaries outside an AFB zone were collected in the scope of the annual national decree on monitoring of animal health status, animal disease eradication programs and vaccinations of animals. This order includes the inspection of apiaries intended for queen bee rearing for the presence of *P. larvae* and other bee pathogens.

### 2.2. Validation of qPCR for the Quantification of P. larvae in Adult Bee Samples

For qPCR quantification of *P. larvae* in hive debris, we used our recently developed qPCR assay [8], which has already been validated for hive debris and honey samples. Its limit of detection (LOD) for hive debris is 188 cells per mL of debris, equaling 0.9 targets per qPCR, and the limit of quantification (LOQ) is 707 cells per mL of debris, equaling 3.5 targets per qPCR. Here, we extended the validation of the assay to adult bee samples. For this, 50 adult bees from each AFB-affected colony were transferred to a filter bag, supplemented with 20 mL of sterile dH_2_O and homogenized (MiniMix; Interscience, Saint Nom, France) at the highest speed for 1.5 min. DNA was extracted from three 1-mL aliquots (representing three biological replicates) using the iHelix Complex kit (Institute of Metagenomics and Microbial Technologies, Ljubljana, Slovenia; https://www.ihelix.eu/ [accessed on 14 August 2024]) according to the manufacturer’s instructions. All three biological replicates underwent *P. larvae* quantification using dPCR, which was used to determine the absolute number of targets (equaling *P. larvae* cells) per µL of the extracted DNA, enabling dPCR-based calibration of qPCR quantification. The dPCR reaction mix contained 7.5 µL of 2× master mix (QuantStudio 3D Digital PCR Master Mix v2; Applied Biosystems by Thermo Fisher Scientific, Foster City, CA, USA), 300 nM of both primers and 200 nM of probe, 3 µL of template DNA and PCR-grade water to a final volume of 15 µL [8]. The dPCR reactions were loaded onto QuantStudio 3D Digital PCR 20K Chips v2 and amplified on a ProFlex 2× Flat PCR System (Applied Biosystems by Thermo Fisher Scientific, Foster City, CA, USA) according to the manufacturer’s instructions. The following amplification protocol was employed: 96 °C for 10 min, 39 cycles of 60 °C for 2 min and 98 °C for 30 s, and 60 °C for 2 min. For qPCR, DNA extracted from the three biological replicates was diluted in a 5-fold dilution series and all dilutions were analyzed in three technical replicates, resulting in nine qPCR results per dilution. The qPCR reaction mix contained 10 µL of 2× master mix (Maxima Probe/ROX qPCR MasterMix; Thermo Fisher Scientific, Waltham, MA, USA), 0.12 µL of passive reference dye ROX (diluted 1:10), 300 nM of both primers and 200 nM of probe, 5 µL of template DNA and PCR-grade water to a final volume of 20 µL. The qPCR reactions were amplified on the 7500 Fast Real-Time PCR System (Applied Biosystems by Thermo Fisher Scientific, Foster City, CA, USA). The following amplification protocol was used: 50 °C for 2 min, 95 °C for 10 min, and 45 cycles of 95 °C for 15 s and 60 °C for 1 min. For the calculation of a linear regression equation of the standard curve, data within the linear dynamic range were considered and LOQ, LOD, Cq cut-off value and qPCR efficiency were determined [8]. Since all three biological replicates were quantified with dPCR and analyzed with qPCR, the obtained Cq values could be converted into the number of *P. larvae* cells per sample unit when considering all dilutions of the standard curve from sample preparation to PCR as previously described [8].

### 2.3. qPCR Quantification of P. larvae in Adult Bee and Hive Debris Samples

**Figure 2:**
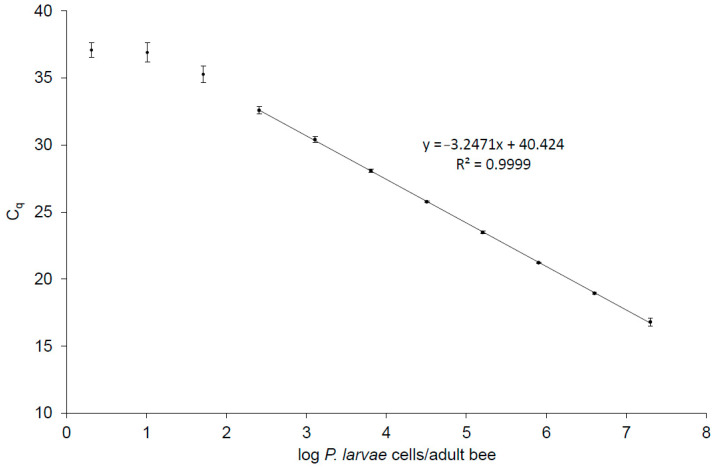
The qPCR standard curve for the quantification of *Paenibacillus larvae* in adult bees. Each of the three biological replicates per dilution was measured in three technical replicates, giving a total of nine measurements per dilution. Dots represent average values and error bars represent standard deviations. The standard curve equation and R^2^ are shown on the graph. Dilutions outside the linear range are also shown.

qPCR quantification of *P. larvae* in hive debris samples (5 mL) was performed as described previously [8]. For qPCR quantification of *P. larvae* in adult bee samples, all samples (50 adult bees per sample) were processed as described in Section 2.2. *P. larvae* cells per sample unit (honeybee or milliliter of hive debris) were calculated from Cq values according to the standard curve equation obtained for adult bee (Figure 2) or hive debris [8] samples, respectively. Positive control (i.e., DNA extracted from a naturally contaminated honey sample that tested positive by bacteriological examination and qPCR in the preliminary analysis) and water (no template control) were included in each qPCR run.

### 2.4. Statistical Analysis

All statistical analyses were performed using GraphPad Prism v9.5.1 (GraphPad Software, San Diego, CA, USA; https://www.graphpad.com [accessed on 1 July 2024]), except the ROC analysis, which was performed using the easyROC v1.3.1 web tool [21]. A *p* value of ≤0.05 was considered statistically significant for all analyses. For the purpose of statistical analyses, all values below the LOQ were set at 0.5 LOQ. Kruskal–Wallis test was used to compare *P. larvae* counts in bee colonies with different severity of AFB clinical signs. Spearman’s correlation was used to assess the correlation between *P. larvae* counts in adult bees and hive debris collected in a paired-sample design. Binary logistic regression and ROC analysis were used to define the optimal cut-off points (expressed as *P. larvae* count) and diagnostic value of both sample types.

## 3. Results

### 3.1. Validation of qPCR for the Quantification of P. larvae in Adult Bee Samples

LOD of the qPCR assay for adult bee samples was 42 cells per adult bee, equaling 3.5 targets per qPCR, and LOQ was 263 cells per adult bee, equaling 21.9 targets per qPCR. The Cq cut-off value was 38.0. The qPCR efficiency was 103.2%, suggesting the absence of PCR inhibitors. The linear dynamic range spanned across seven log_10_ concentrations. The qPCR standard curve is shown in Figure 2.

### 3.2. P. larvae Counts in Colonies with Varying Degrees of Disease Severity

**Figure 3:**
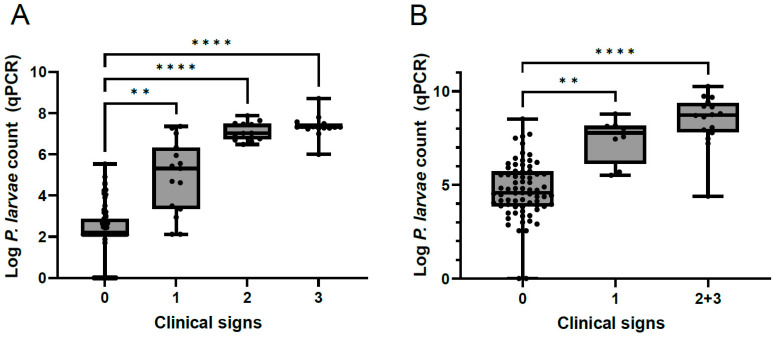
*Paenibacillus larvae* count in adult bees (**A**) and hive debris (**B**) samples determined using qPCR. The samples are stratified according to the severity of AFB clinical signs in the corresponding bee colony: 0, absence of disease; 1–3, presence of AFB clinical signs ranging from the mildest (1) to the most severe (3). The analysis was performed on a complete set of adult bee (*n* = 106) and hive debris (*n* = 97) samples. The groups that differed significantly in *P. larvae* count (as determined by Kruskal–Wallis test) are indicated by asterisks: **, *p* ≤ 0.01; ****, *p* ≤ 0.0001.

*P. larvae* counts were significantly lower in AFB-unaffected colonies (i.e., colonies without AFB clinical signs) than in AFB-affected colonies (i.e., colonies with AFB clinical signs) in both sample types examined (Figure 3; Kruskal–Wallis test, *p* < 0.0014 for adult bees and *p* < 0.0015 for hive debris). The AFB-affected colonies with varying degrees of disease severity did not differ significantly with regard to *P. larvae* counts in any of the sample types examined (Figure 3; Kruskal–Wallis test, *p* > 0.0926). The correlation between *P. larvae* counts in adult bee and hive debris samples (paired samples, *n* = 66) was very strong (Spearman’s correlation coefficient r_s_ = 0.8460; *p* < 0.0001).

*P. larvae* counts in adult bee and hive debris samples from AFB-unaffected colonies outside an AFB zone were either negative (*n* = 11) or below the LOQ (*n* = 2) (Table S1). *P. larvae* counts in samples from AFB-unaffected colonies within an AFB zone ranged from 0 to 3.38 × 10^5^ cells/adult bee and from 0 to 3.31 × 10^8^ cells/hive debris. These two groups differed significantly with regard to *P. larvae* counts, with higher pathogen load observed in the colonies within an AFB zone (Mann–Whitney U-test, *p* < 0.0001). All clinically affected colonies had *P. larvae* counts above 875 cells/adult bee and above 24,625 cells/mL hive debris, which are both above the LOD and LOQ.

### 3.3. Binary Logistic Regression and ROC Analysis

**Figure 4:**
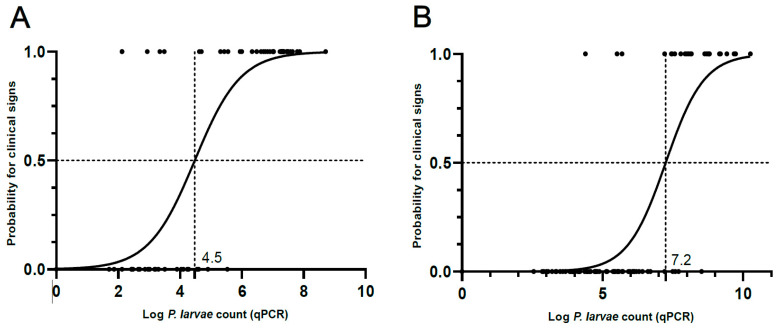
Logistic regression curves for adult bee (**A**) and hive debris (**B**) samples. The curves were constructed on a complete set of adult bee (*n* = 106) and hive debris (*n* = 97) samples. Log *Paenibacillus larvae* count (as determined by qPCR) at a 50% probability of a colony being clinically affected is denoted in both graphs.

**Figure 5:**
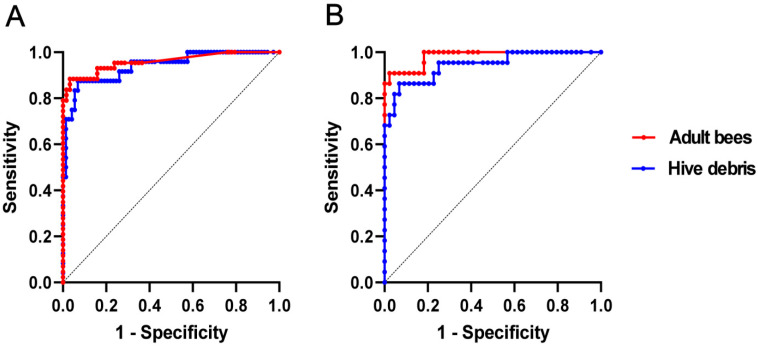
Receiver operating characteristic (ROC) curves for adult bee and hive debris samples. The curves in (**A**) were constructed on a complete set of adult bee (*n* = 106) and hive debris (*n* = 97) samples, whereas the curves in (**B**) were constructed on a subset of 66 paired samples. The curves in (**B**) did not differ significantly (*p* = 0.2506).

The binary logistic regression model for adult bee samples (*n* = 106) revealed that the log *P. larvae* count at a 50% probability of disease occurrence was 4.5 (95% confidence interval from 4.0 to 5.1), equaling 30,000 cells/adult bee (Figure 4A). The area under the ROC curve was 95.9% (Figure 5A). The optimal cut-off based on Youden’s J index for adult bees was 4.6 log cells/adult bee, equaling 42,000 cells/adult bee.

The binary logistic regression model for the hive debris samples (*n* = 97) showed that the log *P. larvae* count at a 50% probability of disease occurrence was 7.2 (95% confidence interval from 6.8 to 7.8), equaling 1.76 × 10^7^ cells/mL hive debris (Figure 4B). The area under the ROC curve was 94.0% (Figure 5A). The optimal cut-off based on the Youden’s J index for adult bees was 7.2, equaling 1.62 × 10^7^ cells/mL hive debris. Performance metrics for both sample types in relation to the ROC analysis confirmed a high diagnostic accuracy of both sample types (Table 2).

To directly compare the diagnostic value of adult bees and hive debris, the ROC curves for both sample types were also constructed on a subset (*n* = 66) of adult bee and hive debris samples collected using a paired-sample design (Figure 5B). The area under the curve (AUC) values for adult bee and hive debris samples were 98.2% and 94.4%, respectively. The AUC areas did not differ significantly (*p* = 0.2506), suggesting that both sample types have a comparably strong diagnostic value.

On average, log_10_ *P. larvae* count per sample unit was 1.6 times higher in the hive debris than in the adult bee samples (paired samples, *n* = 66). Four out of 66 adult bee samples (paired samples) were qPCR-negative; all originated from AFB-unaffected colonies outside an AFB zone (*n* = 1) or within the AFB zone (*n* = 3). Of these, 2/4 samples were qPCR-positive for hive debris, indicating a slightly higher analytical sensitivity of hive debris compared with adult bees.

## 4. Discussion

Reliable methods for the quantification of *P. larvae* in adult bees and other hive samples are necessary for the early detection of high-risk apiaries regarding the onset of AFB. Here, we show that qPCR-based quantification of *P. larvae* in adult bees and seasonal hive debris has a high diagnostic value for predicting the occurrence of AFB disease. qPCR is therefore a fast and cost-effective method for identifying high-risk apiaries, which should undergo thorough clinical examination and targeted sanitary measures to prevent the occurrence and spread of AFB.

Based on the ROC analysis of all and paired samples, the diagnostic value of both sample types was high. Moreover, the ROC analysis of paired samples showed that it did not differ significantly between the two sample types. This confirms the well-established good diagnostic value of adult bees for AFB detection [6,7,11,13,14,16] and extends it to seasonal hive debris, which has been rarely investigated using qPCR quantification of *P. larvae* [8]. Of note, direct comparison of the diagnostic accuracy and cut-offs of the qPCR assay used in this study with other qPCR assays for the quantification of *P. larvae* [14,22,23,24,25] is not possible due to the use of different methods for sample preparation, DNA extraction and qPCR.

The qPCR standard curve for the quantification of *P. larvae* in adult bees showed good performance parameters in terms of PCR efficiency, linear dynamic range, LOD and LOQ. The LOD values for adult bees (this study) and hive debris [8] were 3.5 and 0.9 target copies per qPCR, respectively, and were therefore close to the theoretical LOD of 1–3 target copies per qPCR [20]. Two out of four paired samples from clinically unaffected colonies were qPCR-positive only for hive debris but qPCR-negative for adult bees. In addition, *P. larvae* count per sample unit was generally higher in hive debris than in adult bees. Taken together, the qPCR assay for the quantification of *P. larvae* used in this study seems to have a slightly higher analytical sensitivity in hive debris samples compared with adult bee samples.

Different hive sample types also vary in their accessibility over different seasons and invasiveness. Collecting hive debris is completely non-invasive and easy to perform, and winter hive debris can generally be sampled without opening the hive. However, collecting enough seasonal hive debris requires placing collection boards at the bottom of the hives since hive debris is regularly removed from the hive due to bee hygienic behavior. It should also be noted that only seasonal hive debris was analyzed in this study and therefore, the diagnostic value of winter debris was not assessed.

Sampling of adult bees is relatively non-invasive and easy to perform. Adult bees are easily collected throughout the beekeeping season and at instances when AFB clinical signs are identified and/or not enough hive debris is present in the hive. They are also believed to better predict the current health status of the hive, contrary to *P. larvae* load in winter hive debris which represents the accumulation of spores over time [14].

Although setting a fixed and generally applicable threshold (in terms of *P. larvae* count) to predict the presence of AFB is tempting, the multifactorial nature of AFB, high variability in *P. larvae* count within clinically unaffected colonies and differences in the methodology used make the use of such a threshold difficult. Rather, a relationship between *P. larvae* count and the corresponding probability of the occurrence of AFB disease should be established for each method and each sample type, as exemplified in this study and in previous studies [7,8,14]. Here, we developed a logistic regression model, which maps the observed values onto a scale of disease probability. In addition, ROC analysis was performed, which plots true positive rate versus false positive rate across a range of cut-offs and enables the determination of the optimal threshold in terms of sensitivity and specificity, e.g., based on Youden’s J index. Both methods therefore enable the identification of the optimal specificity/sensitivity ratio.

In this study, the colonies with 4.5 log cells/adult bee and 7.3 log cells/mL hive debris had a 50% probability of disease occurrence. Both thresholds were highly similar to the Youden’s J index-based cut-off values of 4.6 log cells/adult bee and 7.2 log cells/mL hive debris and therefore represent the optimal sensitivity/specificity pairs. These thresholds can therefore be used to identify high-risk apiaries using the methodology described here. We advise that when high-risk colonies with *P. larvae* counts exceeding any of these thresholds are identified, additional sanitary measures should be implemented in such colonies. First, increased clinical examination of such colonies and all the colonies (apiaries) maintained by the same beekeeper should be performed, and in case of detection of colonies with a developed clinical form of the disease, the outbreak must be eradicated in accordance with the regulations for the control of AFB. Second, old combs should be replaced with new foundations (shook swarm) in all asymptomatic colonies with elevated *P. larvae* counts. Third, special emphasis should be put on thorough cleaning and disinfection of beekeeping equipment, strict control of honeybee transfer and clinical examination of colonies. These biosecurity measures are also in line with the established good beekeeping practices and should be applied in addition to the other beekeeping practices that promote bee health and productivity [18]. Molecular quantification of *P. larvae* in bee-related samples, especially adult bees, could provide a basis for the selection of high-risk colonies that should undergo clinical examination since unguided clinical examination of all colonies within the AFB zone is cumbersome. However, the usefulness of molecular quantification of *P. larvae* also depends on the cost of the test and the ease of submitting samples. In Slovenia, official sampling is performed by veterinarians specialized in bee health who work under the umbrella of the National Veterinary Institute. This allows for a centralized and harmonized system for collecting and analyzing samples, which may not exist in other countries.

*P. larvae* counts in AFB-affected colonies with varying degrees of disease severity did not differ significantly, regardless of the sample type tested. This confirms our previous findings that *P. larvae* count in seasonal hive debris from AFB-affected colonies does not differ significantly with varying degrees of disease severity [8]. The lack of such association indicates that AFB is a multifactorial disease whose severity is not only influenced by the *P. larvae* count. Several biotic and abiotic factors are thought to influence the relationship between spore count and clinical signs of AFB. First, *P. larvae* genotypes differ markedly in their virulence. ERIC I genotype exhibits a slow-killing phenotype and is more virulent at the level of bee colony, whereas ERIC II genotype is a fast killer and is less virulent at the colony level [26,27]. Because ERIC II genotype kills most of the brood at the larval stage (before cell capping), the number of dead larvae is higher than in infections with ERIC I genotype, where the proportion of brood that dies after cell capping (at the pupal stage) is higher [26,27]. Consequently, nurse bees can more easily recognize and remove the infected/dead larvae from the uncapped cells in infections with ERIC II genotype, resulting in a lower number of altered comb cells than in infections with ERIC I genotype [27]. In addition, bee-related factors such as host genetics, suppression of individual and social immunity (including hygienic behavior) and host microbiota have also been linked to AFB susceptibility [28,29,30,31]. It is also worth noting that the detection of AFB depends on the beekeeper’s ability to recognize its clinical signs and their willingness to report them to regional bee health experts.

A limitation of the present study is that only a limited number of colonies outside an AFB zone were analyzed. Nevertheless, adult bees from AFB-unaffected colonies within an AFB zone had a significantly higher *P. larvae* count than those from AFB-unaffected colonies outside the AFB zone, which were all below the LOQ. This indicates that the location of the apiary (within vs. outside an AFB zone) significantly influences *P. larvae* counts and that apiaries within the AFB zone have an increased risk of disease onset. Similarly, a previous study using a metagenomic approach showed that adult bees from AFB-unaffected apiaries have lower *P. larvae* counts than those from AFB-unaffected apiaries within an AFB zone [32]. It should also be noted that the apiaries outside an AFB zone were intended for commercial queen bee rearing, which are checked annually for the presence of *P. larvae* and therefore undergo a stricter control program than regular apiaries. *P. larvae* can also be transmitted over large geographic distances through beekeeping activities such as the exchange of beekeeping equipment and colony trade/movement [33], suggesting that AFB control should not rely solely on the examination of hives within an AFB zone.

Since the employed qPCR assay cannot distinguish between live and dead *P. larvae* cells, an overestimation of the pathogen count due to dead cells in hive samples is possible. In the bee host, vegetative *P. larvae* cells are only present in the gut during the early phase of infection. Thereafter, they multiply, cause larval decay and sporulate [1]. Spores are also the only infectious form of *P. larvae*. We can also assume that the conditions outside the host do not favor the survival of vegetative *P. larvae* cells. Therefore, spores were probably the predominant form of *P. larvae* in the samples examined. Furthermore, a slight overestimation of the *P. larvae* count in bee colonies would only positively impact early detection of AFB and its spread by subjecting more apiaries to a thorough clinical field examination, thereby reducing the number of false-negative results.

## 5. Conclusions

In summary, we show here that both the adult bees and the seasonal hive debris have a good diagnostic value for predicting the onset of AFB based on *P. larvae* counts determined by qPCR. Because the AFB-unaffected colonies within an AFB zone had significantly higher *P. larvae* counts than the AFB-unaffected colonies outside an AFB zone, special attention should be paid to the identification of high-risk colonies within an AFB zone and the implementation of appropriate sanitary measures (e.g., shook swarm method).

## Figures and Tables

**Table 1:** The number of analyzed adult bee and hive debris samples with respect to the severity of AFB clinical signs in the corresponding bee colony. The 66 paired samples represented a subset of the total number of adult bee (*n* = 106) and hive debris (*n* = 97) samples. For additional information on the samples, see Table S1.

	AFB-Unaffected Colonies	AFB-Affected Colonies			Sum
**Disease Severity**	**0**	**1**	**2**	**3**	
**Adult Bees (total)**	63	15	13	15	106
**Hive Debris (total)**	73	8	12 *	4 *	97
**Paired Sample Subset (Adult Bees and Hive Debris)**	44	8	11 *	3 *	2 × 66

* These samples were combined into a single group (named ‘2–3’) for the purpose of statistical analysis due to the small number of samples.

**Table 2:** Measures of diagnostic accuracy of the qPCR assay for the quantification of *Paenibacillus larvae* in adult bees and hive debris. The receiver operating characteristic (ROC) analysis was performed using easyROC tool v1.3.1 by applying the Youden’s J index method to determine the optimal cut-off.

Measure	Adult Bees (*n* = 106)	Hive Debris (*n* = 97)
Youden’s J Index (Optimal Criterion)	0.9	0.8
Optimal Cut-Off (log *P. larvae* Count)	4.6	7.2
Sensitivity	0.884	0.875
Specificity	0.968	0.932
Positive Predictive Value	0.950	0.808
Negative predictive Value	0.924	0.958
Positive Likelihood Ratio	27.837	12.775
Negative Likelihood Ratio	0.120	0.134

## Data Availability

All data are available within this article and its Supplementary Materials.

## Supplementary Materials

The following supporting information can be downloaded at: https://www.mdpi.com/article/10.3390/vetsci11090442/s1, Table S1. Sample data.
